# An assessment of quality of home-based HIV counseling and testing performed by lay counselors in a rural sub-district of KwaZulu-Natal, South Africa

**DOI:** 10.1080/17290376.2016.1248477

**Published:** 2016-11-01

**Authors:** Vuyolwethu Magasana, Wanga Zembe, Hanani Tabana, Reshma Naik, Debra Jackson, Sonja Swanevelder, Tanya Doherty

**Affiliations:** ^a^ Health Systems Research Unit, South African Medical Research Council, Francie van Zyl Drive, Parow valley, Tygerberg 7505, South Africa; ^b^ Department of Public Health Sciences, Karolinska Institutet, Stockholm, Sweden; ^c^ School of Public Health, Boston University, 715 Albany Street, Boston, MA 02118, USA; ^d^ School of Public Health, University of the Western Cape, Modderdam Road, Bellville 7535, South Africa; ^e^ Biostatistics Unit, South African Medical Research Council, Francie van Zyl Drive, Parow valley, Tygerberg 7505, South Africa

**Keywords:** home-based HIV counseling and testing, counseling quality, South Africa, lay counselors, supervision, Conseils/tests VIH à domicile, Qualité des conseils, Afrique du Sud, Conseillers volontaires, Supervision

## Abstract

HIV counseling and testing (HCT) has been prioritized as one of the prevention strategies for HIV/AIDS, and promoted as an essential tool in scaling up and improving access to treatment, care and support especially in community settings. Home-based HCT (HBHCT) is a model that has consistently been found to be highly acceptable and has improved HCT coverage and uptake in low- and middle-income countries since 2002. It involves trained lay counselors going door-to-door offering pre-test counseling and providing HCT services to consenting eligible household members. Currently, there are few studies reporting on the quality of HBHCT services offered by lay counselors especially in Sub-Saharan Africa, including South Africa. This is a quantitative descriptive sub-study of a community randomized trial (Good Start HBHCT trial) which describes the quality of HBHCT provided by lay counselors. Quality of HBHCT was measured as scores comparing observed practice to prescribed protocols using direct observation. Data were collected through periodic observations of HCT sessions and exit interviews with clients. Counselor quality scores for pre-test counseling and post-test counseling sessions were created to determine the level of quality. For the client exit interviews a continuous score was created to assess how satisfied the clients were with the counseling session. A total of 196 (3%) observational assessments and 406 (6%) client exit interviews were completed. Overall, median scores for quality of counseling and testing were high for both HIV-negative and HIV-positive clients. For exit interviews all 406 (100%) clients had overall satisfaction with the counseling and testing services they received, however 11% were concerned about the counselor keeping their discussion confidential. Of all 406 clients, 393 (96.8%) intended to recommend the service to other people. In ensuring good quality HCT services, ongoing quality assessments are important to monitor quality of HCT after training.

## Résumé

Le conseil et test VIH ont été priorisés comme une des stratégies de prévention et promus comme un outil essentiel pour le passage à l’échelle et pour l’amélioration de l’accès au traitement et au soutien en particulier au niveau communautaire. Le modèle de conseil et test à domicile a systématiquement été considéré comme acceptable et a depuis 2002 accru sa couverture dans les pays à revenu faible et moyen. Des conseillers volontaires et formés font du porte à porte offrant pré et post test conseil et test VIH aux membres éligibles et consentants des foyers. Il y a actuellement peu d’études sur la qualité des services de conseils et test à domicile offerts par des conseillers volontaires, en particulier en Afrique sub-saharienne, y compris en Afrique du Sud. Nous présentons une sous-étude quantitative d'un essai randomisé au niveau communautaire (Essai de Good Start conseil et test à domicile) qui décrit la qualité des services de conseil et test à domicile offerts par des conseillers. La qualité des services a été mesurée par observation directe utilisant des scores qui reflétaient la pratique observée des conseillers comparée aux protocoles. L’information a été extraite d’observations régulières de sessions de conseil pré et post/test et d’interviews de sortie avec les clients. Pour les clients un score continu a été utilisé pour mesurer leur niveau de satisfaction avec la session. Un total de 196 (3%) sessions ont été observées et 406 (6%) clients ont été interviewes. Globalement, les scores médians de qualité été élevés pour les clients VIH négatifs aussi bien que positifs. Tous les clients interviewes étaient satisfais du service reçu, mais 11% voulaient être assures que le conseiller garderait leur information confidentielle. Des 406 clients, 393 (96,8%) ont affirmés qu’ils recommanderaient ce service à d’autres. Pour assurer le maintien de la qualité des services conseils/tests VIH, il est nécessaire d’avoir une évaluation régulière de la qualité après la formation.

## Introduction

Many developing countries are now providing antiretroviral therapy (ART) for the management and treatment of HIV/AIDS. HIV counseling and testing (HCT) is the entry point that enables access to care and treatment, however uptake remains low in most of sub-Saharan Africa (Matovu & Makumbi, 2007). HCT is also promoted as a cost-effective prevention strategy in most developing countries (Grabbe et al., 2010) because it provides people with the opportunity to receive information on HIV/AIDS and may facilitate reduction of HIV risk behavior (WHO, 2013). Over the last decade various approaches to increase HCT uptake have been implemented including client-initiated HCT, provider-initiated HCT, mobile community-based HCT and home-based HCT (HBHCT) (WHO, 2013).

Client-initiated HCT and provider-initiated HCT are the traditionally known models of HCT and they are usually incorporated into existing health settings (Alcorn & Smart, 2006). Both these models have limitations in terms of coverage (WHO, 2012b). They are often undermined by stigma, poor infrastructure, shortages of skilled service providers and inadequate procurement and supply management systems (WHO/UNAIDS/UNICEF, 2007). On the other hand, provider-initiated HCT has been known to exclude certain groups of people who do not attend or who stay far from health facilities (WHO, 2012b). The HBHCT model was developed as an alternative to these two models, in response to the challenges associated with them.

Mobile community-based HCT and HBHCT models generally target hard-to-reach populations, such as communities residing in rural and historically disadvantaged settings and individuals who have difficulties with accessing health facilities (Yoder, Katahoire, Akol, Bunnell, & Kaharuza, 2006). HBHCT involves trained lay counselors going door-to-door offering pre-test counseling and providing HCT services to consenting eligible household members (WHO, 2012b). It has been consistently found to be highly acceptable and has improved HCT coverage and uptake in low- and middle-income countries since 2002 (Doherty et al., 2013; Matovu, Kigozi, Nalugoda, Wabwire-Mangen, & Gray, 2002). It also saves client time and traveling costs to health facilities (Kyaddondo, Wanyenze, Kinsman, & Hardon, 2012). Use of rapid testing has simplified HBHCT implementation (Clark, Bowles, Song, & Heffelfinger, 2008). HBHCT encourages couple counseling, and thus provides couples and family members with an increased opportunity to learn about one another’s HIV status (WHO, 2012a). Increased knowledge of HIV status among family members could improve attitudes towards the disease and may promote emotional and social support and adherence to medication (Nuwaha, Kasasa, Wana, Muganzi, & Tumwesigye, 2012).

HBHCT is becoming an essential tool in scaling up and improving access to treatment, care and support (UNAIDS, 2010). Relevant to task-shifting initiatives in South Africa, HBHCT allows lay counselors to perform duties that were traditionally performed by professional health workers. Therefore, it is important to evaluate the quality of HBHCT services because poor quality could undermine the benefits of this intervention (Chopra, Doherty, Jackson, & Ashworth, 2005) which are to improve prevention, linkage to care, treatment and support. Previous studies related to HBHCT have largely focused on determining acceptability and monitoring of HCT uptake (Sabapathy, Van der Berg, Filders, Hayes, & Ford, 2012). They report on acceptance, coverage and access to care (UNAIDS, 2010; WHO, 2011), with very few studies reporting on quality of HCT services (Levey & Wang, 2014; Mayanja, 2012; Ron, Wenjuan, & Oyunbileg, 2009). The objectives of this sub-study were to (1) assess the quality of home-based counseling through direct observation of counseling sessions by supervisors comparing observed practice to prescribed protocols and (2) describe client perceptions of the counseling through client exit interviews.

These studies reported concerning levels of underperformance in HCT services due to lack of training, supervision and support (Levey & Wang, 2014; Mayanja, 2012; Ron et al., 2009). A study done in Zambia reported that less than one-third of clients received counseling on reducing number of sexual partners and only approximately 5% of clients received counseling on disclosure of HIV test results to the partners (Levey & Wang, 2014; Ron et al., 2009). The 2012 WHO guidelines on service delivery approaches to HCT, support the adoption of effective and innovative combination of HCT models and good quality cost-effective strategies. The expansion of HCT services must adhere to the human rights of the individual tested as well as the ethical principles of HCT guidelines. Rigorous training, supportive supervision and quality assurance are crucial for good quality of HCT.

Franco, Franco, Kumwenda, and Nkhoma (2002) have described methods for assessing quality of provider performance in developing countries. They cite four common assessment methods: observation of provider performance using a checklist; exit interviews with patients or caretakers about provider performance; reviewing patient or health facility records to assess provider performance; and interviews with providers about their performance. For this study of HCT provider performance we used the first two methods. This paper describes results from direct observations with a checklist and exit interviews with clients who received HBHCT conducted by lay counselors in a rural sub-district of KwaZulu-Natal Province.

## Methods

### Setting

The Good Start HBHCT intervention study was implemented from September 2009 to December 2010 in the UMzimkhulu sub-district of Sisonke District in KwaZulu-Natal, where antenatal HIV prevalence in 2011 was reported to be 40% (NDoH, 2012). The UMzimkhulu sub-district population is approximately 243,024, accounting for almost 50% of the district’s population, 91% of which live in rural areas (Statistics South Africa, 2008). The majority (65%) of households are headed by females. Average household income is below R800 ($78) per month and as a result a large proportion (77%) of the households are dependent on social services and grants (Umzimkhulu local Municipality, 2010). A summary of the study is outlined below. Full details of the trial methods are reported elsewhere (Doherty et al., 2013).

### Description of the HBHCT intervention

The Good Start HBHCT Trial was implemented in 16 randomized clusters (8 control and 8 intervention) by frontline field staff consisting of 11 lay counselors, 4 supervisors and 1 nurse supervisor (registered nurse), who was responsible for overall field supervision. Before the intervention started, the Good Start team (site manager, two study assistant managers and study coordinator) engaged in intensive community mobilization with key stakeholders to acquire permission, create awareness and elicit support for the intervention.

### Qualification and selection of counselors

Minimum selection criteria for lay counselors included having 12 years of schooling (Grade 12), residing in selected clusters and having some experience in health or community work. All the candidates underwent selection and interview processes.

### Qualification and selection of supervisors

Minimum selection criteria for supervisors included having 12 years of schooling (Grade 12), residing in selected clusters and having some experience in health or community work. All the candidates underwent selection and interview processes. Supervisors had additional post-Grade 12 qualification; more than 5 years of field experience in public health research were females.

### Training of counselors

On appointment lay counselors completed the two-week nationally accredited HCT training course. As part of the process of preparing lay counselors for door-to-door HBHCT, counselors were trained on how to negotiate entry into the households.

Following the training course, lay counselors spent 4 months doing in-service training within local clinics to gain confidence in HIV counseling and skills in undertaking the testing procedure. Thereafter all the lay counselors underwent a personal assessment by the nurse at the clinic to determine if their skills were of a high enough standard for them to work independently in their communities. Once this review had taken place they began going door-to-door offering HBHCT to consenting adults (18+ years) and also 14–17 year olds with parental/guardian consent.

### Training of supervisors

Supervisors also completed the two-week nationally accredited HCT training course. Furthermore, supervisors were trained to conduct direct observations and exit interviews assessing quality of counseling and testing offered by counselors.

### Intervention and supervision

Lay counselors received ongoing quality assurance and supervisory visits on two levels. Firstly, four field supervisors and the nurse supervisor worked in separate study clusters to conduct client exit interviews on randomly selected households that were visited by lay counselors. The lay counselors did not know which houses would be visited by the supervisors for client exit interviews. Secondly day-to-day monitoring, and supervision and support of counselors were carried out by the field supervisors, who aimed to observe at least one randomly selected counseling session per counselor per week (with the permission of participants).

### Counseling quality sub-study

This was a descriptive sub-study of a community randomized trial (Good Start HBHCT trial) (ISRCTN31271935).

The sub-study reports on data that were collected by supervisors to assess quality of HCT through observations and exit interviews with clients.

### Sample size

A total of 6757 individuals were offered HCT by the Good Start lay counselors between September 2009 and December 2010 and 5086 (75%) individuals agreed to test. Of these 408 clients had an exit interview and 196 counseling sessions were observed. No formal sample size calculations were undertaken to determine the number of counseling observations and exit interviews since these were part of the intervention supervision process and dependent on the work load and other responsibilities of supervisors. All exit interviews and counseling observations were included in the analysis for this quality sub-study.

### Data collection

All four supervisors and the nurse supervisor conducted direct observations and exit interviews assessing quality of HCT offered by lay counselors. During the consent process for HCT, the lay counselor requested permission for the presence of the observer from all household participants. For exit interviews supervisors and the nurse supervisor also requested verbal consent with all the household participants that have been previously visited by the lay counselors and randomly selected for exit interviews.

For observations of counseling sessions a structured counseling observation tool was used. Counseling observations were intended to assess the quality of the HBHCT sessions including appropriateness of lay counselors’ messages, counseling and testing skills and procedures, as well as their interaction with the clients. Separate sections of the tool covered HIV-negative and HIV-positive post-test counseling. Examples of the questions that were part of the counseling observation tool, included:

1. Did the counselor introduce herself?
2. Did the counselor outline the HCT process?
3. Did the counselor discuss the impact of HIV-positive result on clients’ intimate relationship?

For HIV testing, counselors used rapid HIV test kits that were used by district health facilities during the study period – SD Bioline for screening and SENSA for confirmation of HIV-positive results.

Client exit interviews were undertaken at households already visited by counselors and a structured questionnaire was used. Exit interviews were intended to assess client satisfaction with the services. Examples of the questions that were part of the client exit interview tool, included:

1. Did the counselor approach you with a respectful attitude?
2. Did you feel confident about the counselors’ skills and abilities in offering you counseling and HIV testing?
3. Do you intend to recommend this service to others?

Both the counselor observation checklist and the client exit interview questionnaire were adapted from existing HCT quality assurance tools (FHI, 2002; WHO, 2004). The research team made changes to the tools to suit the setting and the context. The counselor observations took place in the clients’ homes and an entire counseling and testing session was observed from start to finish. Coverage of issues in pre-test counseling, testing and post-test counseling sessions were assessed and ticked off on the observation form and later entered into a cell phone-based form.

Both exit interviews and client observation tools had key or common components determining the quality of HBHCT services offered by lay counselors (see Box 1).

Box 1. **Main areas included in the counseling observation and client exit tool.**

• Interpersonal relations (negotiating home entry, introductions, privacy, confidentiality, attentive listening, eye contact and supportive attitude).

• Pre-HIV testing giving information (giving clear information, assessing client’s understanding, disease risk assessment, impact of HIV results).

• Gathering information (assessing client’s HIV knowledge and discussing couple counseling).

• Active planning and preparation (discussing HIV risk, sexual risk behavior change, condom issuing/demonstration, screening for opportunistic infections and benefits of HIV testing).

• HIV testing (obtaining consent for HIV testing, discussing other testing options, rapid HIV testing procedure, meaning of results, window period and partner referral).

• HIV testing procedure (performing appropriate testing procedure including correct reading and interpretation of results and handling of equipment).

• Interaction and rapport with the client (assessing client’s readiness in receiving results, correct interpretation of results, client’s understanding of results, handling clients emotions appropriately, discussing impact of HIV results on client’s life and intimate relationship, process of disclosure, available support and client’s main concerns).

• Post-HIV testing giving information (discussing community support structures, risk reduction and safe sexual behavior, follow-up visits for re-testing, CD4 count tests and ART treatment).

• Proper recording (proper completion of records and necessary forms including recording correct results and HIV testing kits batch numbers).

• Overall satisfaction and recommendation of service (assessment of client satisfaction, provided time to ask questions and raise main concerns, client’s confidence in lay counselor’s responses to questions and concerns raised, counseling and HIV testing skills).

Both data collection tools were pre-loaded on the cell phone with existing logic, range and consistency checks built-in to minimize gaps or missing data. Once the tool was complete the data were immediately uploaded on the computerized web-based system. All data were hosted and managed by this computerized web-based system. The study team also used this system to monitor data and performance of field workers.

### Data analysis

Data were exported from the Good Start mobile researcher database into a Microsoft Excel spread sheet and then into Stata v12 software for analysis. Counselor quality scores for pre-test counseling, testing and post-test counseling sessions were created as continuous variables with possibilities to create dichotomous cut-off points depending on the distributions observed. For the purposes of this study quality is defined according to the standard requirements of HCT which, as mentioned above, included counseling observations for both interpersonal counseling and clinical skills in performing the actual testing procedure. Counselors’ quality scores were determined by the extent to which counselors included components that were expected to be covered in a HCT session.

For continuous scoring of the counseling observation tool all the questions in the tool were counted. All items are coded with 1 = yes the item was done/discussed, 0 = no the item was not done/discussed. The scores of each single item/variable were added up and summed to create a score per counseling observation. For pre-test counseling the score is out of a maximum of 29 items, for the HIV testing procedure the score is out of a maximum of 12 items and for post-test counseling the score is out of a maximum of 33 items.

For the client exit interviews a continuous scoring was created to assess how satisfied clients were with their counseling session based on the questions in the exit interview. For the exit interview survey there were 17 questions (items) to assess client satisfaction with the counselor. A quality score for each survey was computed by summing the values of each individual item. Median scores with first and third quartile estimates are calculated and presented in the tables.

### Ethical permission

The Good Start trial and this sub-study were both approved by the Ethics Committee of the Medical Research Council (EC009-003). The trial is registered with the International Controlled Clinical Trials database (ISRCTN31271935). Participation in the study was voluntarily. For the exit interviews and counseling observations, an information sheet together with verbal informed consent was obtained from each participant in the household. The information sheet explaining details about the study, benefits and risks, the voluntary nature of the study and assurances of confidentiality was provided in Zulu and read to participants. Confidentiality of study participants was strictly maintained with no identifying information collected in the cell phones. Individuals were informed that they could withdraw from any aspect of the study at any time without giving any reasons. For counseling observations the supervisor sat quietly in the room and did not intervene in the counseling and testing session.

## Results

### Characteristics of lay counselors

All counselors who had 12 years of schooling were females and aged between 26 and 44. They were residing in the intervention clusters, spoke the local language and 9 out of 11 had community health worker related working experience of 2–7 years and 2 had no experience. In addition, some counselors had prior training in infant feeding counseling (*n* = 6), HCT (*n* = 1) non-health training (*n* = 2). Some had prior experience either as an infant feeding counselor (*n* = 5), general community health work (*n* = 3) or both (*n* = 1).

### Characteristics of study participants and the sub-group that had counseling sessions assessed

Table 1 provides characteristics of the total sample who accessed HBHCT and those who had counseling sessions observed. This table shows that a total of 6757 individuals were offered HCT by the Good Start lay counselors of which 1896 (28.1%) were males and 4861 (71.9%) were females. Overall 196 (3%) of the full study sample had counseling sessions observed and 406 (6%) had exit interviews. There were similarities in characteristics between the full study sample and the participants that had their counseling sessions assessed. Amongst the full study sample and the sub sample with counseling assessments the majority (71.9% and 82.7%, respectively) were females and (41.7% and 46.8%, respectively) were married.

**Table 1.** Characteristics of all study participants and the sub group that had counseling sessions assessed.

Characteristics	All participants *n* = 6757 (%)	Participants who had their counseling sessions assessed *n* = 196 (%)
Gender		
Male	1896 (28.1)	34 (17.3)
Female	4861 (71.9)	162 (82.7)
Household size		
<3	3621 (53.6)	118 (60.2)
>or equal to 3	3136 (46.4)	78 (39.8)
Marital status		
Single	1929 (37.7)	50 (26.0)
Married	2130 (41.7)	90 (46.8)
Cohabiting	179 (3.5)	8 (4.2)
Widowed	819 (16.0)	42 (22.0)
Divorced/Separated	53 (1.1)	2 (1.0)
Age categories		
14–24	1985 (29.9)	54 (27.7)
25–34	1163 (17.5)	28 (14.4)
35–44	921 (13.8)	12 (6.1)
45 and above	2581 (38.8)	101 (51.8)
Ever tested for HIV		
Yes	2174 (42.6)	93 (48.4)
No	2926 (57.4)	99 (51.6)

### Counselor assessments and quality scores

**Fig 1.**
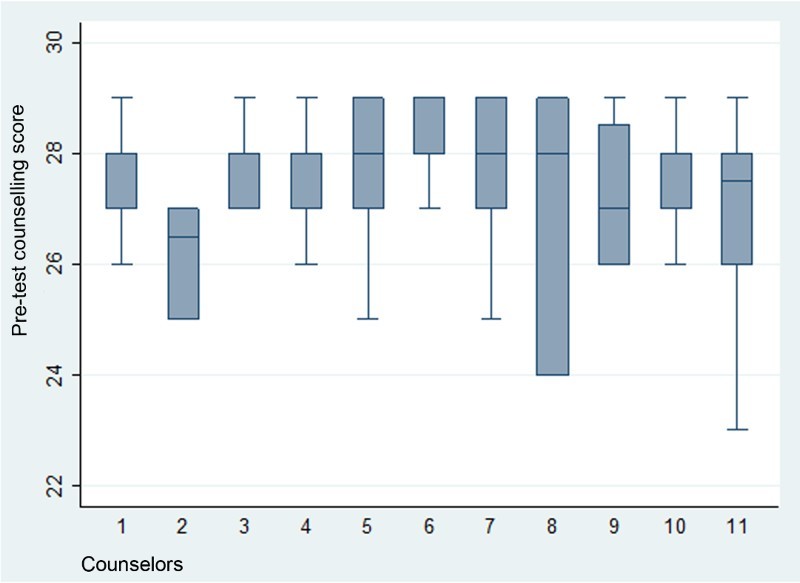
Counseling observations: pre-test counseling scores.

From September 2009 to December 2010, four supervisors and one nursing supervisor conducted 196 counseling observations. The number of counseling observations per supervisor varied from 19 to 65 and the median number of assessments per supervisor was 34. Fig. 1 shows the range and distribution of pre-test counseling quality scores among counselors. The total number of pre-test counseling sessions observed was 196. The number of HCT sessions each lay counselor had observed ranged from 7 to 31. The box for Counselor 8 is relatively long, indicating that her pre-test counseling scores occupy a relatively wide range (24–29). In contrast, Counselors 1, 3, 4, 6 and 10 have compact interquartile ranges (scores ranging from 27 to 28 and 28 to 29), with their pre-test counseling scores concentrated in the higher end of the distribution. One of the counselors’ box plot (Counselor 2) does not have lines extending vertically from the box indicating lack of variability in her pre-test counseling scores outside the upper and lower quartiles, showing that her performance was fairly consistent although it did not reach the highest end of the distribution as was the case for Counselors 5, 6, 7 and 8.

Table 2 shows quality scores for HIV testing and post-test counseling observation sessions. All lay counselors obtained high median scores with the lowest being 26.5 out of 29. Out of 196 counseling sessions observed 192 (98%) HIV testing and only 14 (7%) were HIV positive. For HIV testing sessions observed, all 11 counselors achieved high scores ranging between 9 and 12 and 4 lay counselors achieved 100% compliance with the counseling standards and scored 12/12 for all HIV-testing sessions observed (Counselors 3, 4, 5 and 6), 2 counselors achieved 100% compliance with HIV-negative post-test counseling (Counselors 6 and 7) and another 5 out of the 9 lay counselors who had HIV-positive post-test counseling sessions observed scored 21/21, also achieving 100% compliance with HIV testing standards for post-test counseling (Counselors 1, 4, 5, 7 and 8).

**Table 2.** Quality scores: HIV testing procedure and post-test counseling by HIV status.

Counselor	Total HIV testing sessions observed	Median (IQR) out of a total of 12	Total HIV-negative post-test counseling sessions observed	Median score (IQR) out of a total of 10	Total HIV-positive post-test counseling sessions observed	Median score (IQR) out of a total of 21
1	14	12.0 (10–12)	13	9.0 (9–10)	1	21.0 (21–21)
2	7	12.0 (11–12)	7	10.0 (9–10)	0	0
3	15	12.0 (12–12)	14	9.0 (9–19)	1	19.0 (19–19)
4	24	12.0 (12–12)	23	10.0 (9–10)	1	21.0 (21–21)
5	24	12.0 (12–12)	21	10.0 (9–10)	3	21.0 (21–21)
6	21	12.0 (12–12)	20	10.0 (10–10)	1	20.0 (20–20)
7	7	12.0 (9–12)	6	10.0 (10–10)	1	21.0 (21–21)
8	4	12.0 (10–12)	3	10.0 (9–10)	1	21.0 (21–21)
9	8	12.0 (11–12)	8	10.0 (9–10)	0	0
10	26	12.0 (11–12)	23	10.0 (7–10)	3	21.0 (19–21)
11	29	12.0 (11–12)	28	10.0 (9–10)	1	20.0 (20–20)
Average	16.2/Counselor	Median for the group 12.0 (12–12)	15.0/Counselor	Median for the group 10.0 (9–10)	1.2/Counselor	Median for the group 21.0 (19–21)

Note: Interquartile range, IQR.

### Quality of counseling for certain critical components of HCT

Table 3 shows quality of counseling for certain critical components of HCT. Lay counselors achieved good results for the critical components of HCT.

**Table 3.** Quality of counseling for certain components of HCT.

Critical counseling topics/actions	*N* (%) correct
Finding a private area for counseling	194/196 (98.9)
Discussing confidentiality	192/196 (97.9)
Assess clients risk for HIV	195/196 (99.5)
Screen for TB	190/196 (96.9)
Screen for STIs	185/196 (94.4)
Wearing gloves	191/192 (99.5)
Waiting 20 minutes before reading the screening results from the test strip.	182/192 (94.8)
Discuss sexual behavior change (HIV risk reduction)	171/196 (87.2)
Condom use demonstration	44/196 (22.4)
Discuss disclosure plan for HIV-negative clients	177/178 (99.4)
Discussing safe sex and giving condoms for HIV-negative result	129/177 (77.9)
Discuss disclosure for HIV-positive result	14/14 (100)
Explain the window period	181/181 (100)

### Client exit interviews

**Fig 2.**
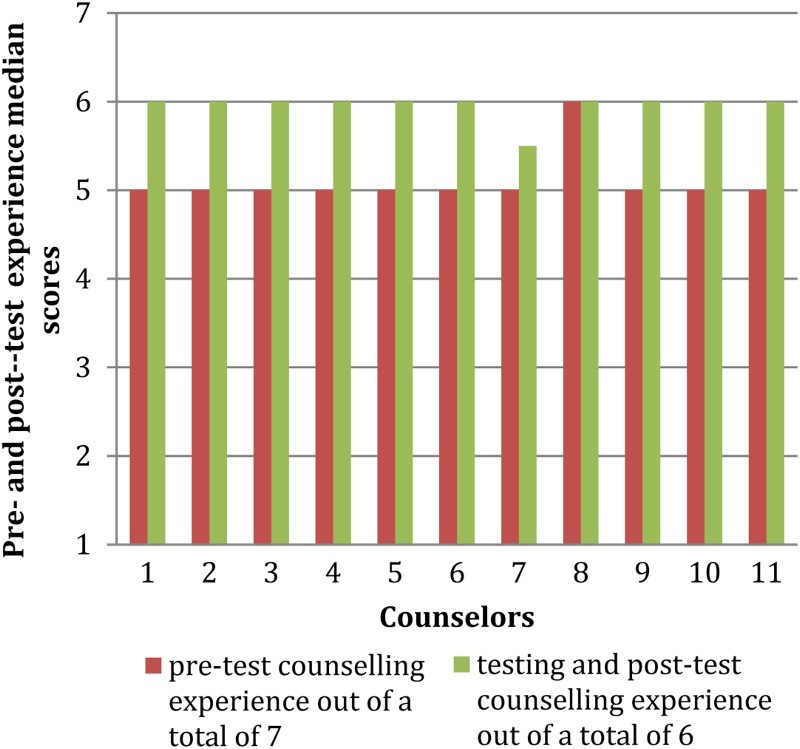
Client exit interviews median scores.

Fig. 2 shows that for pre-test counseling, all lay counselors acquired a score of 5 out of 7 except for 1 (Counselor 8) who scored 6 indicating that clients were satisfied with the quality of counseling they received for certain key aspects such as respect, confidence in their skills and confidentiality. For HIV testing and post-test counseling, all counselors except one scored a median of 6 out of 6 indicating high level of satisfaction with the HIV testing procedure and post-test counseling.

Out of 406 client exit interviews conducted, all clients reported that lay counselors introduced themselves and explained the purpose of their visit to the household well, and all except one said that the counselor approached them with a respectful attitude. The majority (71.6%) of clients interviewed had agreed to be tested by the lay counselors in their home and of these 287 (98.9%) were comfortable with the HIV testing procedure and all except 1 (99.6%) were comfortable with the manner in which results were explained. However, 33 (11.3%) were concerned about counselors keeping their discussion confidential. Of the 291 clients who were tested 272 (93%) were informed about other available services and 268 (97.5%) of these clients were offered follow-up counseling and lay counselors clearly explained the follow-up procedure. All 406 clients that had exit interviews, had overall satisfaction with the HCT they received, and were confident about lay counselor’s skills and abilities in offering counseling and HIV testing. Of all 406 clients, 393 (96.8%) intended to recommend the service to other people.

## Discussion

To our knowledge this is the first study to report on the quality of HBHCT services offered by lay counselors in South Africa. This paper has reported high-quality scores for pre-test counseling HIV testing and post-test counseling by lay counselors for both HIV-positive and HIV-negative clients. The results also show that overall, clients were satisfied with the quality of counseling offered by lay counselors. The remarkable performance of several counselors who achieved 100% compliance with HIV testing and post-test counseling standards is indicative of the adequate training and supportive supervision the counselors received.

A publication by Jackson et al. (2013) based on the same study sample reported that of the 3986 dried blood specimens taken by these lay counselors, 99.4% of rapid test results matched with the laboratory gold standard results. The laboratory sub-study found high sensitivity and specificity of 98% (95% CI: 96.3–98.9%) and 99.6% (95% CI: 99.4–99.7%), respectively. Another paper from this study assessing acceptability of HBHCT, reported 75% HBHCT uptake (Naik, Tabana, Doherty, Zembe, & Jackson, 2012). Taken together, these sub-studies, including this paper, display a positive picture about the performance of these lay counselors, not only in terms of high uptake of HIV testing, but also in high quality of actual HIV testing. This performance indicates that lay counselors can render HCT services which were traditionally the role of professional health workers and this may ease the burden on the health systems.

The finding of high-quality HCT in our study may be due to the fact that this was done in the context of a research intervention where lay counselors received a high level of training on both theory and practice followed by mentoring and supportive supervisory visits. After the initial 2 weeks training, lay counselors spent 4 months doing practical in-service training in the local clinics to gain confidence in counseling and improve skills in the HIV testing procedure. This was followed by personal assessments conducted by supervisors to determine if lay counselor’s skills were of a high standard to work independently in their communities. During these assessments lay counselors received feedback concerning their level of performance, which highlighted what they were doing well, as well as what they needed to improve. Haines et al. (2007) suggest that high quality of services provided by lay health workers is dependent on adequate training, supervision and systems support. This is evident with similar studies assessing quality of HCT in Uganda. Results of a study conducted in Kampala District, clearly demonstrated that quality of HCT was compromised due to lack of HCT service providers who had ever undergone in-service training in HCT. Only 148 (73%) of the 204 HCT providers ever received in-service training in HCT. This study also reported that there was minimal supportive supervision from the district or partners, only 40 (39%) of the 101 HCT providers were ever supervised , moreover facilities lacked standard operating procedures and guidelines and there were few qualified HCT service providers especially in the area of counseling (Mayanja, 2012). Another HBHCT study conducted in Brazil also reported more discrepancies on the interpretation of HIV and syphilis test results by nurses compared to nurse practitioners, and this was associated with the nurse practitioners level of experience and skills in using rapid test kits for malaria and dengue (Ribeiro et al., 2015). A hospital-based study done in Uganda also reported that the health workers missed important opportunities for HIV/AIDS prevention education with their patients, approximately 26% of health workers had never referred patients for HIV counseling and 31% had never advised patients who were at risk of HIV infection to be tested. Reason for this poor performance included time constrains and/or lack of related knowledge or skills (Mungherera et al., 1997).

It is also important to highlight that the intervention had a very high supervisor to counselor ratio with 5 supervisors (4 counselor supervisors and 1 professional nurse) to 11 lay counselors. This ratio might not be feasible in a scaled-up national program. As this study was the first of its kind in rural South Africa, it needed high levels of supervision to prove whether it is safe and effective before scaling it up. For national implementation, CHWs should be linked to local health facilities for ongoing training and supervision.

Although the study found a very high performance by lay counselors, 11% of clients who had exit interviews were not confident that lay counselors would keep their discussion confidential. Other studies have reported similar findings (Bategenya, Abdulwadud, & Kiene, 2007), confirming, that clients may sometimes struggle to trust that lay counselors, who are from the same communities as their clients, will keep their discussion or HIV test results confidential, and this could potentially affect HCT uptake. However, we also believe that recruiting lay health workers within the area facilitated effective communication with stakeholders and clients. Furthermore, 3% of clients expressed that they would not recommend the service to other people. This study could not ascertain the reasons for these concerns.

In 78% of counseling observations, the counselor did not offer demonstration of condom use. This could be due to the fact that during the intervention, lay counselors asked clients if they knew how to use a condom and if they said yes, then the counselor did not offer to demonstrate condom use. Cultural practices and lack of privacy in the households may also have had an effect on condom demonstration during counseling. Also, lay counselors may have felt uncomfortable or inappropriate to do condom demonstrations for married or elderly clients who were our dominant population. While this could be appropriate for this rural setting, low scores for condom demonstration could impact negatively on sexual behavior change and this therefore highlights the importance of strong linkages to health facilities and other services offering counseling on primary and secondary prevention of HIV/AIDS.

## Limitations

This study was limited in that we only used a quantitative approach to measure quality of counseling and client perceptions of the service. A qualitative approach could have provided a greater breadth and depth of information, particularly concerning clients’ emotions and views related to HCT. Furthermore, qualitative interviews with the counselors to explore their experiences of performing HBHCT would have been informative. This should be considered for similar studies in the future.

Each counselor was expected to have an observation of their counseling every week, but due to other demands on supervisors, these assessments were not performed as frequently as planned. Resulting in only 3% of the total number of clients (6757) accessing HBHCT having their counseling session observed. Similarly, the sample of clients who participated in an exit interview was small and data on their socio-demographics were not collected. As a result other clients who received the service but were not randomly selected for exit interviews may have had different views of the counseling experience. However, the consistency in the results of both the observations and exit interviews (i.e. that both were very high) is reassuring.

The sample of counseling observations amongst HIV-positive clients was small (7%), however this sample is similar to the HIV prevalence of 8% reported within the whole study (Good Start trial) sample (Doherty et al., 2013). Caution should be exercised when generalizing these results since they represent the quality of counseling amongst mainly HIV-negative clients.

## Conclusion

HCT has been prioritized as a strategy for prevention and access to care, treatment and support, expanding HCT services at community level may reduce the workload at health facilities and is in line with current task-shifting strategies for community-based health care services (Schneider & Lehmann, 2010). The findings of this paper demonstrate that it is feasible for lay counselors to provide good quality HBHCT in a rural setting.
